# Supplementary material for: Impact of Aging and Visual Input on Postural Stability in Dogs: Insights from Center-of-Pressure Analysis
Source: Sensors (Basel). 2025 Feb 20;25(5):1300. doi: 10.3390/s25051300 (PMC11902305; doi:10.3390/s25051300)
Supplement: Supplementary file 1 [file sensors-25-01300-s001.zip › sensors-3468602-supplementary.pdf]

Table S1. Descriptive statistics (mean ± SD) of the base of support (BOS), its length (L) and width (W) of adult (G1) and senior (G2) dogs during sighted (EO) and blindfolded (EC) measurements.

Condition	Group	BOS (cm^2)	BOS L (cm)	BOS W (cm)
EO	G1	1055.00 ± 204.23	53.35 ± 6.93	19.96 ± 2.70
	G2	979.69 ± 196.59	51.16 ± 5.95	19.50 ± 2.79
EC	G1	1025.02 ± 226.56	52.53 ± 6.05	19.65 ± 2.85
	G2	994.06 ± 252.32	51.37 ± 6.93	19.69 ± 3.99

Table S2. P-values of the comparison of group 1 (adult dogs) and group 2 (senior dogs) during sighted (EO) and blindfolded (EC) measurements for the base of support (BOS), its length (L) and width (W).

Condition	BOS (cm^2)	BOS L (cm)	BOS W (cm)
EO	0.242	0.290	0.594
EC	0.685	0.577	0.967

Table S3. P-values of comparison of the base of support (BOS), its length (L) and width (W) during sighted (EO) and blindfolded (EC) in group 1 (adult dogs) and group 2 (senior dogs).

Group	BOS (cm^2)	BOS L (cm)	BOS W (cm)
G1	0.669	0.690	0.750
G2	0.838	0.918	0.844
